# Enhanced High-Temperature Corrosion Resistance of AISI 301LN Stainless Steel in Silica-Doped Ternary Carbonate Nanofluids for Thermal Energy Storage Applications

**DOI:** 10.3390/ma19153283

**Published:** 2026-08-03

**Authors:** Miguel Morales, Mohammad Rezayat, Antonio Mateo

**Affiliations:** 1 CIEFMA, Department of Materials Science and Engineering, EEBE—Campus Diagonal Besòs, Universitat Politècnica de Catalunya—BarcelonaTech, C/Eduard Maristany 16, 08019 Barcelona, Spain; mohammad.rezayat@upc.edu (M.R.); antonio.manuel.mateo@upc.edu (A.M.); 2 Barcelona Research Center in Multiscale Science and Engineering, EEBE—Campus Diagonal Besòs, Universitat Politècnica de Catalunya—BarcelonaTech, C/Eduard Maristany 16, 08019 Barcelona, Spain

**Keywords:** molten salts, silica nanoparticles, high-temperature corrosion resistance, anticorrosion mechanism, stainless steel

## Abstract

**Highlights:**

- SiO_2_ nanofluids reduced AISI 301LN corrosion by up to 60%.
- SiO_2_ doping increased oxide-scale hardness and protectiveness.
- <20 nm SiO_2_ doping showed more protective barrier than <50 nm SiO_2_.
- Si-rich reticulated nanostructures formed within oxide scales.
- A corrosion protection mechanism in SiO_2_ salt nanofluids is proposed.

**Abstract:**

Molten carbonate salt nanofluids have emerged as a promising approach to improve the power generation efficiency of next-generation concentrated solar power (CSP) systems due to their enhanced thermophysical properties at high temperatures. However, corrosion upon salt nanofluids remains a key challenge for the use of cost-effective steels as construction materials in CSP applications. In this work, the corrosion behavior of AISI 301LN stainless steel exposed to molten carbonate salt nanofluids containing 1.0 wt.% SiO_2_ nanoparticles with <20 nm and <50 nm has been studied. Corrosion tests were conducted in a static Li_2_CO_3_-Na_2_CO_3_-K_2_CO_3_ molten salt mixture at 600 °C for 1000 h. The oxide scales formed after exposure to the three nanofluids and the base salt were compared. The results revealed that the corrosion rate of AISI 301LN steel on molten salt was reduced by the addition of SiO_2_ nanoparticles. The incorporation of SiO_2_ nanoparticles into the oxide scale leads to the formation of dense reticulated nanostructures composed of Si-containing oxides, respectively. This increases the hardness of the oxide scale and enhances its protective performance in molten salt, particularly when using SiO_2_ nanoparticles with the smallest size (<20 nm).

## 1. Introduction

Addressing the current energy crisis requires efficient energy storage solutions to manage the intermittency of renewable energy sources such as solar and wind. Thermal Energy Storage (TES) offers high efficiency, low operational costs, and scalability [1,2,3,4,5]. Commonly used in Concentrated Solar Power (CSP) plants, TES systems typically include a storage medium, a heat transfer mechanism, and a containment system [6,7,8]. While nitrate molten salts are standard in commercial CSP applications [9,10,11], next-generation technologies are focusing on carbonate and chloride salts due to their superior high-temperature performance and energy efficiency potential [12,13]. Carbonate salts, in particular, provide favorable thermophysical properties and lower corrosivity toward CSP materials [14,15,16,17], though challenges remain due to their high melting point (~400 °C) and cost [18,19,20]. Among these, the eutectic LiNaK carbonate mixture (32.1 wt.% Li_2_CO_3_; 33.4 wt.% Na_2_CO_3_; 34.5 wt.% K_2_CO_3_) stands out for its optimal performance and cost-effectiveness [21,22].

Previous research has established the high-temperature compatibility of various austenitic and duplex stainless steels (e.g., AISI 304, 316, 2205) with eutectic carbonate melts, typically forming Li-mixed oxide scales [22,23,24,25,26,27,28]. Given that CSP plants are designed to operate for over 30 years, extensive research has focused on corrosion mitigation to limit degradation to a few tens of micrometers per year [21,22]. Recent strategies using carbonate and nitrate salts include the following: (1) adding nanoparticles to molten salts [29], (2) applying protective oxide coatings [30,31,32,33,34,35,36], and (3) surface texturing of stainless steels via fractal [37] and laser methods [38,39,40,41,42,43]. Among these, nanoparticle-doped molten salts, also known as molten salt nanofluids (NFs), have shown particular promise. This approach not only reduce corrosion in carbon and stainless steels but also enhances specific heat capacity and thermal conductivity. The nanoparticles help stabilize the corrosion layer, thereby significantly decreasing corrosion rates in nitrate salt environments [29]. In this regard, Fernández et al. [44] reported an exceptionally low corrosion rate of 0.007 mm yr^−1^ for AISI 347 stainless steel in solar salt containing 1 wt.% Al_2_O_3_ nanoparticles, attributed to the formation of a protective Al_2_O_3_-enriched layer. Similarly, Nithiyanantham et al. [45] showed that adding 1 wt.% Al_2_O_3_ or SiO_2_ nanoparticles reduced the corrosion rate of A516 Gr70 carbon steel by more than 60% compared with pure solar salt. Moreover, Gao et al. [46] also demonstrated that SiO_2_ and TiO_2_ nanoparticles enhance passive film stability and reduce corrosion kinetics in molten nitrate salts. Under CSP-relevant dynamic conditions, Nieto-Maestre et al. [47] reported negligible corrosion of AISI 347 stainless steel and confirmed the beneficial effect of Al_2_O_3_ nanoparticles, while subsequent studies [48,49] showed that nanoparticle addition reduces corrosion layer thickness and improves molten salt stability. Thus, these studies highlight the key role of nanoparticles in stabilizing protective oxide layers and enhancing corrosion resistance.

Nanoparticle doping can mitigate corrosion in CSP thermal storage systems by promoting protective layer formation and improving salt properties. However, adverse effects have also been reported. Grosu et al. [50] and Piquot et al. [51] found that nanoparticles increased the corrosion of A516 Gr70 carbon steel in HitecXL molten salt at 310 °C by modifying the corrosion mechanism. Al_2_O_3_ and SiO_2_ nanoparticles produced corrosion layers 2–3 times thicker than in the base salt due to air microbubble entrapment, which increased local oxygen levels. At 390 °C, the bubbles disappeared and corrosion rates became lower than in pure salt, despite nanoparticle agglomeration during prolonged exposure [50].

Nanoparticle-enhanced carbonate salts exhibit higher heat capacity than nitrate salts when using identical nanoparticles, with reported specific heat enhancements of 10–35% for carbonates compared to 5–15% for nitrates [29,52,53,54,55,56]. Recent studies have confirmed improved specific heat and thermal conductivity in carbonate molten-salt nanofluids, which are strongly dependent on nanoparticle type, dispersion quality, and operating temperature [57,58,59,60,61,62,63,64]. An optimal nanoparticle concentration of around 1 wt.% is widely reported [58,59,60,61,62,63,64,65], as higher loadings often cause viscosity increases of 20–50%, offsetting thermal benefits. Even so, data on the corrosion inhibition of steels in carbonate salts with nanoparticles remain scarce. Schuller et al. [66] reported that adding 1.0 wt.% Al_2_O_3_ or SiO_2_ to molten Li_2_CO_3_-K_2_CO_3_ halved the corrosion rate of AISI 304 at 520 °C. Similarly, Frangini and Loreti [67] found that CaO and MgO nanoparticles reduced corrosion of 316L stainless steel in eutectic Li–Na carbonate at 650 °C, with benefits up to 10 mol%. Yang et al. [68] observed corrosion reductions of 18.7%, 25.8%, and 6.5% for three different stainless steel grades, AISI 201, SS304, and SS316L, respectively, in carbonate salts with 1.0 wt.% ZnO (30 nm) at 550 °C, attributing improvements to nanoparticle migration into the corrosion scale. Grosu et al. [56] showed that ternary carbonate nanofluids with 12 nm Al_2_O_3_ at 600 °C halved AISI 310 corrosion and improved heat capacity (~7%), thermal conductivity (~12%), and viscosity (~35%). Furthermore, Fernandez et al. [69] reported enhanced corrosion resistance in alumina-forming austenitic alloys (OC4, HR224) at 650 °C. More recently, Morales et al. [70] demonstrated that doping AISI 301LN steel with ~1 wt.% Al_2_O_3_ nanoparticles in Li_2_CO_3_–Na_2_CO_3_–K_2_CO_3_ at 600 °C reduced the corrosion rate by close to 50%. Those findings demonstrated improved corrosion resistance linked to the presence of Al_2_O_3_, underscoring the potential of nanoparticle-reinforced salts and alloys for high-temperature applications.

The aim of this work is to evaluate the corrosive behavior at 600 °C of austenitic stainless steel 301LN in contact with two eutectic carbonate molten-salt-based nanofluids, obtained by doping with <20 nm and <50 nm SiO_2_ nanoparticles, using the most commonly reported nanoparticle concentration reported in the literature (1 wt.%). SiO_2_ nanoparticles exhibit an interesting chemical behavior in molten carbonate salts due to their acidic character, promoting the formation of Fe_2_SiO_4_ and a Si-rich reticulated nanostructure that may enhance the mechanical integrity and protective performance of the oxide scale [57,58,59,60,61,62,63,64,65]. Previous works have pointed out AISI 301LN and duplex stainless steels as promising alloys for certain components of CSP systems exposed to molten carbonate salts [24,25]. AISI 301LN offers an interesting balance between corrosion and mechanical resistance with lower cost than other 300-series steels [71,72,73] and its fatigue and wear resistance can be further enhanced by surface treatments [74,75,76,77]. In the present work, samples were tested by immersion in a static Li_2_CO_3_, Na_2_CO_3_, and K_2_CO_3_ mixture with SiO_2_ nanoparticles at 600 °C for 1000 h. Compositional, microstructural, and micromechanical analyses of oxide scales formed in the base salt and nanofluid environments were conducted to elucidate the underlying corrosion mechanisms and assess their mitigation as a function of nanoparticle size and the nature of the two different nanoparticles.

## 2. Materials and Methods

The AISI 301LN stainless steel composition is shown in Table 1. Specimens (50 × 20 × 2 mm^3^) were ground with 1200-grit SiC paper, cleaned ultrasonically in ethanol, and weighed.

Three molten carbonate salt systems were prepared using a dry method according to previous studies [38,39,40,41,42]: a base salt and two nanofluids containing <50 nm and <20 nm SiO_2_ nanoparticles. A eutectic composition (32 wt.% Li_2_CO_3_—33 wt.% Na_2_CO_3_—35 wt.% K_2_CO_3_) was selected due to its low melting point (397 °C), suitable for thermal energy storage. Carbonate salts (99% purity) and nanoparticles were supplied by Sigma-Aldrich (St. Louis, MO, USA). Prior to weighing, the salts were dried at 180 °C for 48 h. Nanofluids were prepared by mixing 99 wt.% salt with 1 wt.% nanoparticles for 20 min, while the base salt was processed under the same conditions without nanoparticles.

Corrosion tests were conducted by immersing samples in molten salts contained in alumina crucibles under air at 600 °C, above the salt melting point (397 °C), to simulate operating conditions of molten-salt phase change materials in CSP systems. After exposure, the furnace was cooled to 450 °C, and samples were removed, rinsed with deionized water, and dried. Corrosion rates were determined using the mass-loss descaling method in accordance with ASTM G1-03 [78]. Surface oxides were removed by immersing the samples for 1–3 min in a 10% (*v*/*v*) HCl solution, followed by rinsing and weighing with a high-precision microbalance, following previous studies [38,39,40,41,42]. The corrosion rate (CR) was calculated from mass loss using Equation (1):

(1) $$CR=\frac{8760\cdot ∆m}{\rho \cdot t}$$

where Δ*m* is the mass change per unit initial surface area (mg/cm^2^), *ρ* is the material density (g/cm^3^), *t* is the exposure time (h), and 8760 represents the number of hours in one year. Reported values represent the average of at least three specimens. The microstructure of oxide scales and corrosion products was examined by field emission scanning electron microscopy (FESEM) coupled with EDS (Carl Zeiss Merlin, Berlin, Germany). Surface and cross-sectional analyses were performed. Cross-sections were prepared by mounting corroded samples in phenolic resin, followed by grinding with 600–1200 grit SiC papers and polishing with 30, 6, and 3 μm diamond suspensions. Crystalline phases were identified by XRD (Bruker D8-Advance, Bruker, Billerica, MA, USA) using Cu Kα radiation (40 kV, 40 mA). Measurements were carried out at room temperature over a 2θ range of 30–80° with a step size of 0.01° and 1 s per step. Phase identification was performed using the JCPDS database and DIFFRACplus EVA software. Hardness measurements of AISI 301LN and the oxide scale near the oxide–steel interface were conducted using a Nanoindenter XP (Agilent Technologies, Santa Clara, CA, USA) with a Berkovich tip. Hardness values were calculated using the Oliver and Pharr method [79]. Samples were embedded, ground, and polished as previously described [80]. Two test series were performed per sample: 40 indents for the substrate and 20 indents for the oxide scale, using a maximum displacement of 500 nm and spacings of 20 μm and 10 μm, respectively. Finally, Google Gemini 3.6 software was used to assist in the development of the conceptual graphical representation of the corrosion mitigation mechanism.

## 3. Results and Discussion

### 3.1. Corrosion Assessment

**Figure 1:**
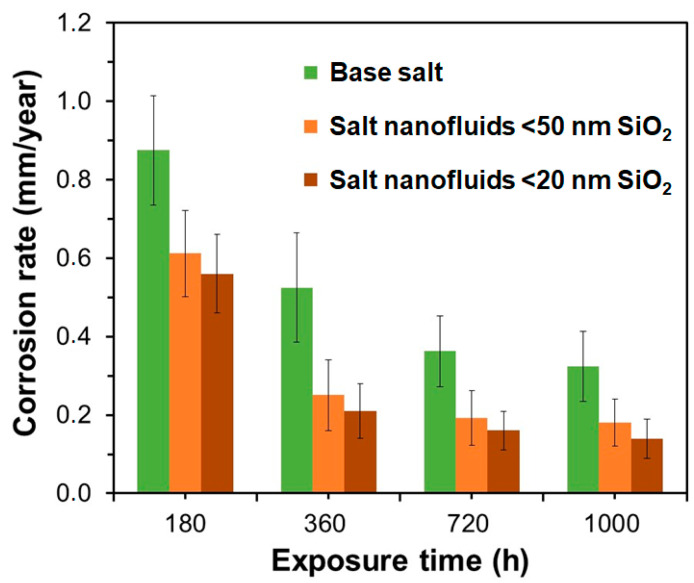
Evolution of corrosion rates for AISI 301LN in base salt and salt nanofluids with <20 nm and <50 nm SiO_2_ nanoparticles at 600 °C determined from the descaling loss method.

In this section, the corrosion assessment of the AISI 301LN stainless steel in molten salt nanofluids is analyzed by the descaled loss method. Figure 1 shows the corrosion rate as a function of exposure time in the base salt and molten salt nanofluids. During the initial 180 h, a significant corrosion rate was observed in all salts. However, after 360 h, the corrosion rate gradually decreased as the exposure time was extended. The progression of the corrosion rate in AISI 301LN is aligned with findings from previous studies on austenitic and duplex stainless steels [25,27,56]. During the initial period of corrosion, the stainless steel undergoes rapid oxidation due to direct contact between the molten salt and the steel surface, leading to intense redox reactions and high the corrosion rates. After a few hundred hours of exposure, the corrosion products substantially hinder the molten salt’s ability to continue reacting with the steel surface. Indeed, the corrosion rates determined at 720 h and 1000 h were remarkably similar for each type of salt. The development of this protective layer governs the corrosion rate in all salts, thereby decreasing it to lower levels. Nevertheless, the corrosion rate of AISI 301LN in both molten salt nanofluids was considerably reduced compared to that in the base salt. After 1000 h, the corrosion rate was 0.33 ± 0.10 mm/year in the base salt, decreasing to 0.18 ± 0.07 mm/year for the salt nanofluids with <50 nm SiO_2_ nanoparticles and to 0.15 ± 0.06 mm/year with the <20 nm SiO_2_ nanoparticles, showing nearly a 50–60% reduction across all salt nanofluids. This phenomenon is ascribed to the addition of nanoparticles into the oxide scale, which improves its protective performance.

### 3.2. Analysis of Oxide Scales

**Figure 2:**
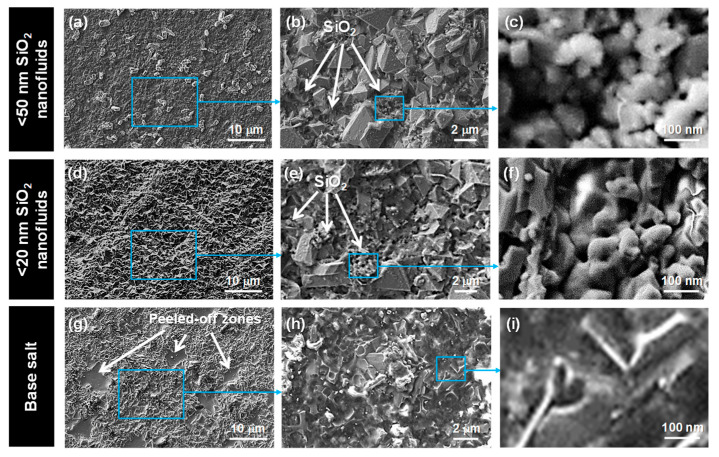
Surface SEM images of AISI 301LN after immersion for 1000 h in salt nanofluids with: (**a**–**c**) <20 nm SiO_2_ nanoparticles and (**d**–**f**) <50 nm SiO_2_ nanoparticles; and (**g**–**i**) base salt.

The compositional and microstructural analyses of the oxide scales formed during corrosion tests were carried out using SEM-EDS and XRD. SEM images of the AISI 301LN samples exposed to molten salt nanofluids and base salt after 1000 h are shown in Figure 2. The addition of SiO_2_ nanoparticles in the salt nanofluids stabilized the formation of the oxide scale, which induced a more uniform oxidation surface (Figure 2a–f). The oxide-scale microstructure formed in the molten salt nanofluids presented enlarged and polyhedral grains (Figure 2b,e). Acicular and reticulated nanostructures of SiO_2_ were grown between the polyhedral grains (Figure 2c,f). In contrast, a considerable density of agglomerated particles was observed on the surface of the oxide scale formed with the base salt (Figure 2g–i). Furthermore, this oxide scale also presented spalled and micro-cracks after the corrosion test. As discussed below, the EDS analyses conducted on the peeled-off regions revealed a higher concentration of Cr than the nominal composition of the oxide scale. This observation indicates that the oxide scale likely has a multilayered structure, with an inner layer enriched in Cr that may be covered by additional oxide layers consisting of different phases.

**Figure 3:**
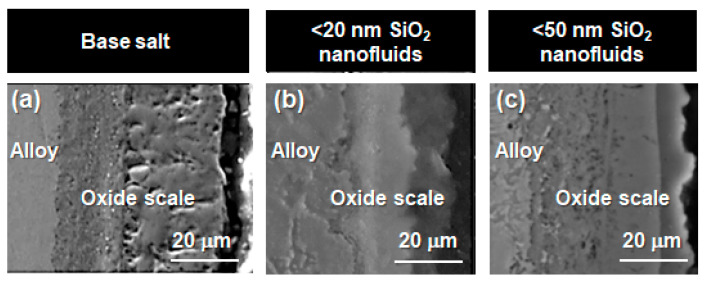
Cross-sectional SEM images of the oxide scales formed on AISI 301LN after 1000 h exposure to: (**a**) base molten salt, and molten salt nanofluids with: (**b**) <20 nm and (**c**) <50 nm SiO_2_ nanoparticles.

The cross-sectional SEM images in Figure 3 illustrate the oxide layers formed on the AISI 301LN surface after exposure to base salt and molten salt nanofluids for 1000 h. Post-corrosion analysis revealed that the oxide scales were predominantly continuous and compact. However, some defects such as pores, voids, and vertical grooves at the interface between the inner and outer oxide layers were observed (Figure 3a), which are often linked to the formation of LiFeO_2_-containing corrosion products [81,82,83]. Notably, as shown in Figure 3b,c, the oxide layers formed on the samples immersed in molten salt nanofluids exhibited a remarkably lower concentration of such defects compared to those exposed to the base salt. Furthermore, the oxide scale thickness on AISI 301LN immersed in molten salts containing SiO_2_ nanoparticles was significantly reduced compared to that in the pure molten salt. In addition, at the end of the corrosion tests, the base molten salt exhibited a considerably more intense coloration compared with the SiO_2_-containing molten salt nanofluids, suggesting a higher concentration of dissolved metallic species generated during the corrosion process and the subsequent formation and dissolution of metal oxide compounds. This finding indicates a more pronounced corrosion mechanism in the base salt environment, characterized by increased metal dissolution and oxide formation. The higher amount of corrosion-derived species detected in the base salt further suggests that the oxide scale formed during exposure was likely more extensive than that observed after testing, as part of the oxide layer may have been removed due to dissolution, spallation, or chemical interaction with the molten salt.

**Figure 4:**
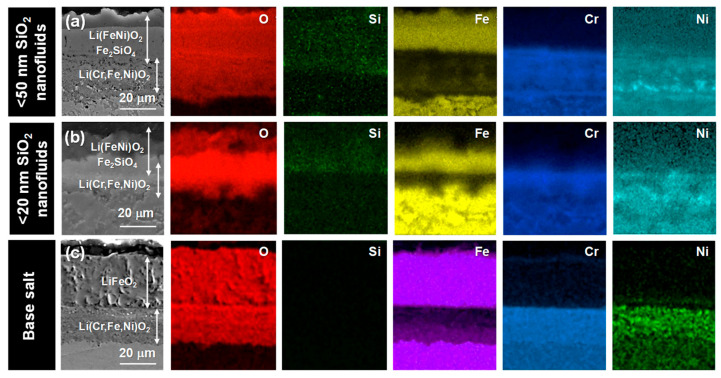
Cross-sectional SEM images and EDS mapping of the oxide scales formed on AISI 301LN after 1000 h exposure to molten salt nanofluids with: (**a**) <50 nm, (**b**) <20 nm SiO_2_ nanoparticles, and (**c**) base molten salt.

Figure 4 presents cross-sectional SEM images alongside EDS elemental maps showing the oxide layers formed on stainless steel surfaces after 1000 h of exposure to both base molten salt and salt nanofluids. The corrosion process resulted in a characteristic multilayered oxide structure for all samples, but with some differences in the distribution of alloying elements. The scales formed on the samples immersed in salt nanofluids displayed significant incorporation of Si-containing oxides, along with a remarkable enrichment of Ni in the outer oxide layer (Figure 4a,b). In contrast, as shown in Figure 4c, the outer layer of the sample immersed in the base molten salt was predominantly enriched in Fe, whereas the inner layer contained higher concentrations of Cr and Ni. It is well established that the incorporation of Cr and Ni oxides into these oxide layers significantly enhances corrosion resistance by forming effective barriers that hinder further oxidation. Therefore, the synergistic effect associated with SiO_2_ addition suggests that salt nanofluids promoting the enrichment of Cr- and Ni-rich scales may provide superior protective performance compared with the base molten salt.

**Figure 5:**
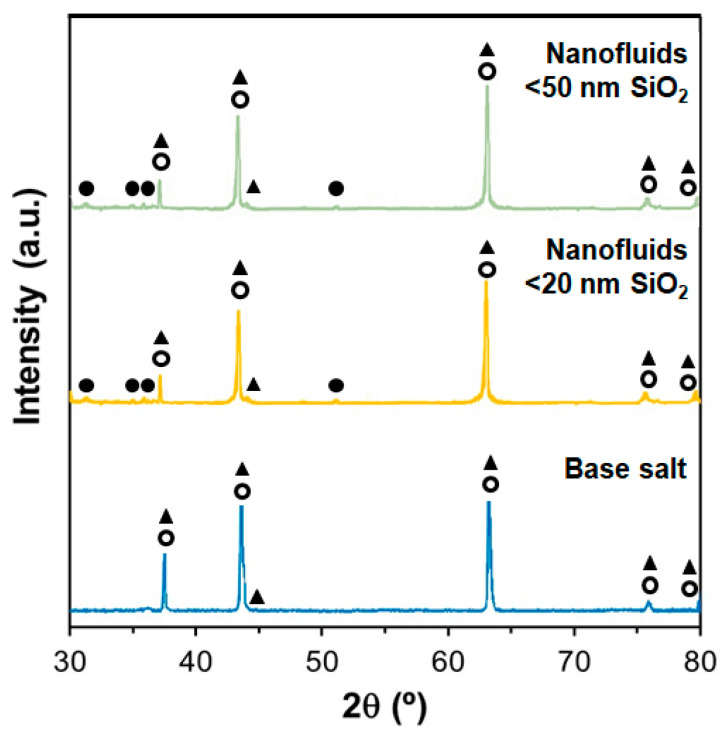
Top-surface XRD spectra of the pristine AISI 301LN and after 1000 h exposure to base molten salt and molten salt nanofluids. The symbols denote the phases: ▲ Li(Cr,Fe,Ni)O_2_; ○ LiFeO_2_ and Li(Fe,Ni)O_2_; ● Fe_2_SiO_4_.

Figure 5 displays the XRD spectra of the reference AISI 301LN after 1000 h exposure to base molten salt and molten salt nanofluids. For the sample exposed to the base molten salt, the predominant corrosion products are lithium ferrites, such as LiFeO_2_, Li(Fe,Ni)O_2_ and Li(Cr,Fe,Ni)O_2_. These phases were identified through complementary SEM–EDS and XRD analyses (Figure 4 and Figure 5). Their formation is characteristic of stainless steels exposed to Li-containing molten salts and arises from the outward diffusion and selective oxidation of Fe, Ni, and Cr, followed by subsequent reactions with lithium species present in the molten salt [56,84]. The relatively sharp and intense ferrite-related peaks suggest the formation of a continuous but chemically complex oxide scale. LiFeO_2_ was predominantly formed in the Fe-rich outer layer, whereas Li(Cr,Fe,Ni)O_2_ was developed within the Cr/Ni-rich inner layer. Doping with SiO_2_ nanoparticles led to the appearance of new peaks attributed to Fe_2_SiO_4_. These results suggest that SiO_2_ nanoparticles do not remain chemically stable within the molten salt matrix and that the corrosion products undergo further chemical reactions. For both <20 nm and <50 nm SiO_2_ nanoparticle-containing nanofluids, Fe_2_SiO_4_ phase was identified, accompanied by lithium ferrites. The presence of the silicates demonstrates that SiO_2_ nanoparticles dissolved or reacted within the molten salt environment and subsequently participated in corrosion product formation. Compared with the base salt, the relative intensity of lithium ferrite peaks was reduced, particularly for the <20 nm SiO_2_ nanofluids, suggesting a partial suppression of Fe-rich oxide formation. This behavior was attributed to the development of Si-rich oxide phases that acted as diffusion barriers for Fe and Cr cations. Furthermore, the differences observed between the nanofluids containing <20 nm and <50 nm SiO_2_ nanoparticles indicate a particle-size-dependent effect on phase evolution. The finer nanoparticles promote stronger silicate formation, consistent with their higher surface area and enhanced reactivity in the molten salt. This results in a more complex but potentially more protective corrosion scale. Overall, the XRD results indicate that nanoparticle additions significantly modify the corrosion-induced phase evolution of AISI 301LN in molten salts. Whereas the base salt primarily promotes lithium ferrite formation, SiO_2_ nanofluids lead to the formation of iron silicates with implications for oxide scale morphology and transport properties. In the nanofluids, the inner and outer oxide layers exhibit higher Cr and Ni contents, respectively, compared with the base salt, contributing to a reduced corrosion rate through stabilization of the oxide scale via nanoparticle–substrate interactions, in agreement with previous studies [56]. No adverse effects related to air microbubbles, such as porosity or scale defects, were observed, likely due to the high operating temperature of the molten carbonate salts.

### 3.3. Nanoindentation Tests of the Oxide Scales

**Figure 6:**
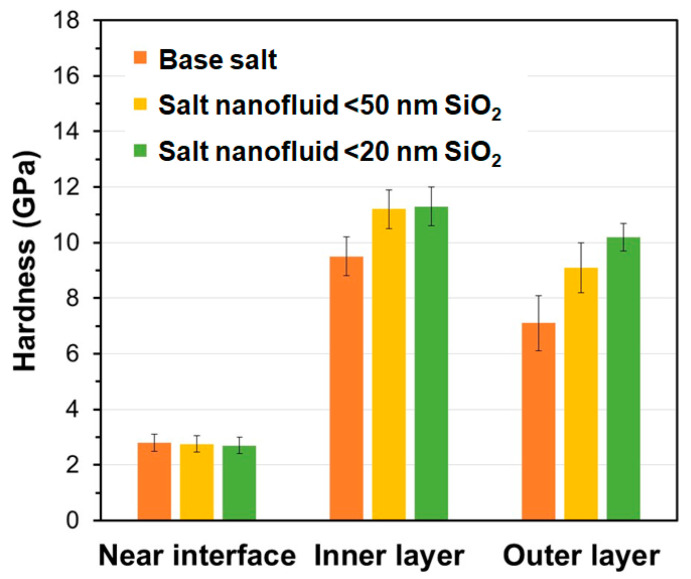
Hardness at the bulk, near the oxide-steel interface, and at the inner and the outer layers of the oxide scales formed on AISI 301LN after 1000 h exposure to base molten salt, and molten salt nanofluids with <50 nm and <20 nm SiO_2_ nanoparticles.

The nanoindentation technique was employed to investigate the effect of nanoparticles on the hardness of oxide scales and the related microstructural changes in AISI 301LN steel during corrosion testing in molten salts. In particular, the hardness values were obtained near the oxide-steel interface, and at the inner and the outer layers of the oxide scales after 1000 h of corrosion testing (Figure 6). The hardness of the bulk AISI 301LN steel for all samples after corrosion tests exhibited similar values to those of the as-received material. Quantitatively, this bulk hardness of AISI 301LN was approximately 2.8 ± 0.1 GPa. This value aligns with reported hardness ranges for other AISI 300 series austenitic stainless steels typically used in concentrated solar power (CSP) applications, such as 310 (2.5–2.7 GPa) [85], 316L (2.8 ± 0.1 GPa) [86,87], and 347 (2.3–2.6 GPa) [88] typically used for CSP applications. Notably, an increase in hardness was observed near the oxide-steel interface, which can be attributed to residual stresses and phase transformations, particularly martensitic transformations, induced by rapid cooling from the furnace to ambient temperature. Therefore, there was no significant change in the overall hardness of the steel after the 1000 h corrosion period, aside from localized increases near the oxide interface. In comparison, the oxide layers formed in the presence of salt nanofluids demonstrated a notable increase in hardness, approximately 30–40% higher than those formed in the base salt. This enhancement is consistent with the denser and more compact oxide morphology observed by SEM, which exhibited a substantially lower density of pores, voids, and interfacial defects. The chemical composition and phase distribution within the oxide scales remained largely similar across different conditions, with the exception of the incorporation of Si-containing oxide phases in the nanofluid samples. The incorporation of these Si-containing phases promoted the development of a mechanically more robust oxide scale. Previous studies have shown that harder and denser oxide scales exhibit greater resistance to crack initiation and spallation, thereby preserving the integrity of the protective barrier and limiting the formation of diffusion paths for oxidizing species and metallic cations [66,89,90]. Consequently, the enhanced hardness observed in the nanofluid samples reflects improved mechanical integrity and barrier effectiveness, contributing to superior corrosion resistance. Therefore, assessing the hardness via nanoindentation offers valuable insights into the quality and protective efficacy of oxide scales formed during high-temperature corrosion processes, complementing the microstructural observations and confirming that the addition of SiO_2_ nanoparticles contributes to improved oxide-scale integrity and long-term corrosion protection.

### 3.4. Mechanism of Corrosion Mitigation

**Figure 7:**
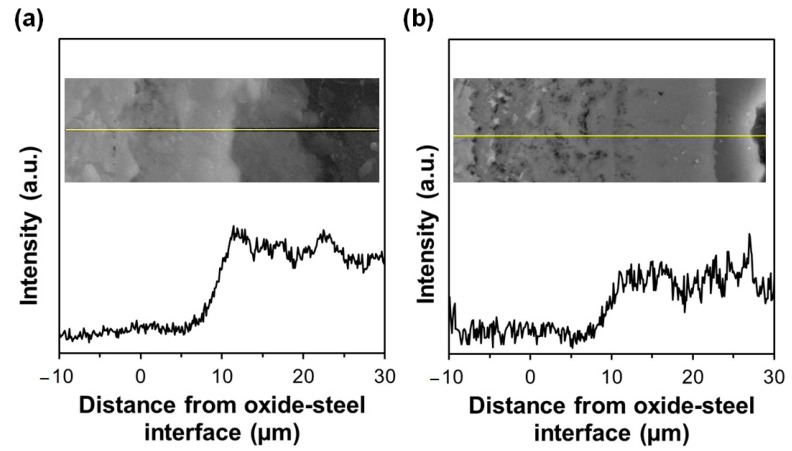
Concentration profiles (in normalized arbitrary units) across the oxide-steel interface obtained from EDS line scans for the Si using: (**a**) <20 nm SiO_2_, and (**b**) <50 nm SiO_2_ nanoparticles for AISI 301LN after a 1000 h corrosion test at 600 °C under salt nanofluids. The inset SEM micrographs show the scanned line.

The incorporation of SiO_2_ nanoparticles into the oxide scale is influenced by particle size and provides a mechanistic explanation for the improved corrosion resistance of AISI 301LN stainless steel exposed to molten salt-based nanofluids. To complement the XRD and SEM–EDS elemental mapping analyses, cross-sectional SEM–EDS line scans were performed to determine the Si distribution across the oxide scale. Figure 7 presents the Si concentration profiles measured after 1000 h of exposure at 600 °C. In both cases, Si enrichment is detected within the oxide scale, particularly toward the outer region, confirming the incorporation of Si derived from the nanoparticles. However, the oxide formed in the nanofluid containing <20 nm SiO_2_ nanoparticles (Figure 7a) exhibits a higher Si concentration and a more continuous profile than that obtained with <50 nm SiO_2_ nanoparticles (Figure 7b), indicating more efficient transport and incorporation of the smaller nanoparticles. The enhanced and more homogeneous Si enrichment promoted by the smaller nanoparticles is expected to favor the formation of dense, chemically stable mixed oxides that act as effective diffusion barriers to oxygen and aggressive ionic species. This particle-size dependence can also be rationalized by considering the much larger number of nanoparticles and the higher specific surface area provided by the nanofluids containing <20 nm SiO_2_ nanoparticles at the same weight fraction. The increased interfacial area enhances nanoparticle/molten-salt interactions and promotes faster interfacial reactions, leading to a more efficient incorporation of Si-containing species into the growing oxide scale. This improved barrier effect is consistent with the lower corrosion rates observed for the nanofluids containing <20 nm SiO_2_ nanoparticles, highlighting the beneficial role of nanoparticle size in promoting protective oxide scale development.

From the analytical results, several key observations can be made:

(1) The surface concentration of Si-containing oxide phases is notably high, well above the XRD detection threshold, indicating substantial incorporation of Si species into the oxide structure.
(2) The oxide scale comprises a complex mixture of Cr-, Fe-, and Ni-rich oxides interspersed with Si-containing oxide phases, forming a reticulated nanostructure that enhances the mechanical stability and barrier properties of the oxide scale.
(3) The outer oxide layer exhibits a significantly higher Si concentration compared to the inner layer, suggesting that SiO_2_ nanoparticles or their compound derivatives preferentially participate in the outer-scale formation through reaction or adsorption mechanisms at the molten-salt/oxide interface.

These findings are consistent with the surface chemistry of SiO_2_ in molten carbonate media. According to the Lux–Flood acid–base concept, SiO_2_ behaves as an acidic oxide capable of reacting with oxide ions (O^2−^) present in oxide-rich carbonate melts to form silicate species. These species subsequently react with Fe-rich oxides within the corrosion scale, promoting the formation of thermodynamically stable Fe–Si mixed oxides, such as Fe_2_SiO_4_, thereby increasing the chemical stability and compactness of the protective oxide layer.

**Figure 8:**
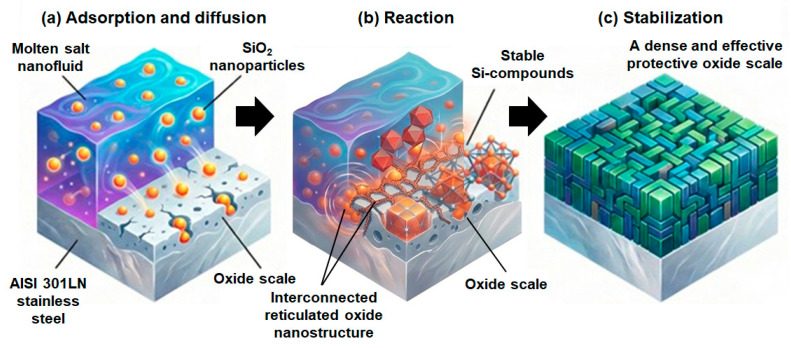
Schematic illustration of the oxide-scale formation mechanism on the AISI 301LN surface after 1000 h exposure to molten salt nanofluids, indicating the main mechanistic steps: (**a**) adsorption and diffusion of the nanoparticles, (**b**) reaction, and (**c**) stabilization of the oxide scale, and elucidating the role of SiO_2_ nanoparticles in corrosion mitigation.

All the above-mentioned observations suggest that SiO_2_ nanoparticles actively participate in the corrosion process through both physical incorporation and chemical interaction with the oxide scale, rather than behaving as inert fillers. Figure 8 summarizes the main mechanistic steps, explaining the incorporation and the positive effect of the SiO_2_ nanoparticles into the protective oxide scale:

(1) Adsorption/diffusion step: SiO_2_ nanoparticles diffuse from the molten salt toward the oxide surface driven by concentration and chemical potential gradients. Upon arrival, these preferentially adsorb at high-energy sites such as defects, pores, and grain boundaries in the outer oxide layer. The adsorption is thermodynamically favored by surface energy minimization and occurs through interfacial interactions ranging from weak van der Waals forces to stronger electrostatic or ionic bonding, depending on the molten salt and oxide chemistry.
(2) Reaction step: At high temperature, the silicate species generated from SiO_2_ react with pre-existing Li-oxides (e.g., Li_2_O or Li_2_CO_3_) and Fe-rich oxides at the oxide-steel interface, leading to the formation of thermodynamically stable Fe–Si mixed oxides (e.g., Fe_2_SiO_4_). The larger specific surface area of the <20 nm nanoparticles accelerates these interfacial reactions, promoting the development of an interconnected, reticulated oxide nanostructure that strengthens and stabilizes the outer oxide scale.
(3) Stabilization step: The newly formed complex oxide layer, consisting of Fe_2_SiO_4_ and mixed phases for SiO_2_ nanofluids, progressively stabilizes into a dense, interwoven structure. This stabilized network remarkably reduces oxygen permeability through the oxide scale, acting as an effective protective barrier that limits further interaction with both oxygen and the molten salts. Therefore, the dissolution of metal oxides (e.g., LiFeO_2_, Li(Fe,Ni)O_2_, and Li(Cr,Fe,Ni)O_2_) within the scale is significantly inhibited, thereby substantially enhancing overall corrosion resistance.

These results indicate that nanoparticle incorporation proceeds through a synergistic interplay of diffusion, precipitation, and chemical reactions for SiO_2_ nanoparticles. This mechanistic incorporation not only modifies the microstructural characteristics of the oxide scale but also enhances its protective action in aggressive molten salt environments. This mechanistic interpretation explains the superior corrosion resistance observed for the nanofluid with <20 nm SiO_2_ nanoparticles, whose higher particle density and larger specific surface area promote more efficient interfacial reactions and the formation of compact Fe–Si mixed oxides. The relative contribution of each step in the mechanism is governed by both the exposure temperature and the alloy composition. These findings are in agreement with recent studies [61,62,63,64,65,70], which highlighted that surface modifications and nanoparticle additions can synergistically stabilize passive oxide films in aggressive carbonate environments. Some of these steps are common to other oxide-scale formation mechanisms in molten-salt environments. For carbon steels tested at low temperatures, the processes of diffusion [91] and precipitation [66,92] played an important role in the inclusion of nanoparticles into the oxide layer. In addition to these mechanisms, for stainless steels in high-temperature nanofluids, the nanoparticles in molten salt reacted to produce stable mixed oxides [49,56,93].

## 4. Conclusions

The corrosion resistance of AISI 301LN stainless steel for thermal energy storage applications was investigated by immersing the alloy in three eutectic carbonate molten-salt-based nanofluids, obtained by doping with SiO_2_ nanoparticles (<20 nm and <50 nm), using the most commonly reported nanoparticle concentration in the literature (1 wt.%), at 600 °C for 1000 h. The main conclusions are summarized as follows:

(1) The carbonate molten salt nanofluids with SiO_2_ nanoparticles significantly enhanced the corrosion resistance of AISI 301LN. The corrosion rate was reduced from 0.32 ± 0.09 mm/year in the base molten salt to 0.18 ± 0.07 mm/year for the salt nanofluids with <50 nm SiO_2_ nanoparticles, and 0.15 ± 0.06 mm/year for <20 nm SiO_2_ nanoparticles, showing nearly 50–60% reduction across all salt nanofluids.
(2) Microstructural, chemical and mechanical analyses revealed remarkable differences between the oxide scales formed in nanofluids and those developed in the base molten salt. In the later one, the oxide scale consisted primarily of Li(Cr,Fe,Ni)O_2_ in the inner layer and LiFeO_2_ in the outer layer. In contrast, the oxide scales formed in SiO_2_ molten salt nanofluids contained significant amounts of Si-containing phases, mainly Fe_2_SiO_4_, which were concentrated in the outer layer. These observations suggest that the presence of the Si-containing oxides in the outer oxide scale induced the formation of Li(Fe,Ni)O_2_ and decreased defects such as pores and voids. Consistent with this interpretation, the hardness of the outer oxide scale increased by ~30–40%, improving its mechanical integrity.
(3) Based on the experimental observations, the corrosion mitigation mechanism in SiO_2_-doped molten salts is proposed to involve to the formation of acicular and reticular nanostructures composed of Si-containing oxides and corrosion products. These structures may contribute to stabilizing and densifying the oxide scale, thereby enhancing its compactness and protective effectiveness and potentially limiting the outward diffusion of Cr and Ni from the substrate and the peel-off of the oxide scale.
(4) Molten salt nanofluids containing SiO_2_ nanoparticles smaller than 20 nm developed a slightly harder outer oxide layer compared with those incorporating <50 nm SiO_2_ nanoparticles, which is consistent with a lower defect concentration (e.g., pores and voids), suggesting a slightly more effective corrosion mitigation mechanism and superior protective performance.

## Figures and Tables

**Table 1:** Chemical composition of AISI 301LN (wt.%) [70].

Alloy	Fe	Cr	Ni	Mn	C	N	Si	P	S
**AISI 301LN**	Bal.	17.6	7.2	1.2	0.02	0.12	<1.0	<0.03	<0.006

## Data Availability

The original contributions presented in this study are included in the article. Further inquiries can be directed to the corresponding author.

## References

[1] Liu M., Tay N.H.S., Bell S., Belusko M., Jacob R., Will G., Saman W., Bruno F. (2016). Review on concentrating solar power plants and new developments in high temperature thermal energy storage technologies. Renew. Sustain. Energy Rev..

[2] Lovegrove K., Csiro W.S., Lovegrove K., Stein W. (2012). Introduction to concentrating solar power (CSP) technology. Concentrating Solar Power Technology.

[3] Lovegrove K., Pye J., Lovegrove K., Stein W. (2012). Fundamental principles of concentrating solar power (CSP) systems. Concentrating Solar Power Technology.

[4] Liu M., Saman W., Bruno F. (2012). Review on storage materials and thermal performance enhancement techniques for high temperature phase change thermal storage systems. Renew. Sustain. Energy Rev..

[5] Prieto C., Ruiz-Cabañas J., Madina V., Fernández A.I., Cabeza L.F. (2022). Lessons learned from corrosion of materials with molten salts during molten salt tank preheating. Sol. Energy Mater. Sol. Cells.

[6] Cabeza L.F., Gutierrez A., Barreneche C., Ushak S., Fernández Á.G., Fernádez A.I., Grágeda M. (2015). Lithium in thermal energy storage: A state-of-the-art review. Renew. Sustain. Energy Rev..

[7] Bauer T., Pfleger N., Laing D., Steinmann W.D., Eck M., Kaesche S., Lantelme F., Groult H. (2013). High-Temperature Molten Salts for Solar Power Application. Molten Salts Chemistry.

[8] Reddy R.G. (2011). Molten salts: Thermal energy storage and heat transfer media. J. Phase Equilib. Diffus..

[9] Parrado C., Marzo A., Fuentealba E., Fernández A.G. (2016). 2050 LCOE improvement using new molten salts for thermal energy storage in CSP plants. Renew. Sustain. Energy Rev..

[10] Ruiz-Cabañas F.J., Prieto C., Madina V., Fernández A.I., Cabeza L.F. (2017). Materials selection for thermal energy storage systems in parabolic trough collector solar facilities using high chloride content nitrate salts. Sol. Energy Mater. Sol. Cells.

[11] Ding W., Bauer T. (2021). Progress in Research and Development of Molten Chloride Salt Technology for Next Generation Concentrated Solar Power Plants. Engineering.

[12] Sarvghad M., Delkasar Maher S., Collard D., Tassan M., Will G., Steinberg T.A. (2018). Materials compatibility for the next generation of Concentrated Solar Power plants. Energy Storage Mater..

[13] Gomez J.C. (2011). High-Temperature Phase Change Materials (PCM) Candidates for Thermal Energy Storage (TES) Applications.

[14] Kenisarin M.M. (2010). High-temperature phase change materials for thermal energy storage. Renew. Sustain. Energy Rev..

[15] Myers P.D., Goswami D.Y. (2016). Thermal energy storage using chloride salts and their eutectics. Appl. Therm. Eng..

[16] Fernández A.G., Cabeza L.F. (2020). Corrosion evaluation of eutectic chloride molten salt for new generation of CSP plants. Part 1: Thermal treatment assessment. J. Energy Storage.

[17] Fernández A.G., Cabeza L.F. (2020). Corrosion evaluation of eutectic chloride molten salt for new generation of CSP plants. Part 2: Materials screening performance. J. Energy Storage.

[18] Mehos M., Turchi C., Vidal J., Wagner M., Ma Z., Ho C., Kolb W., Andraka C., Kruizenga A. (2017). Concentrating Solar Power Gen3 Demonstration Roadmap.

[19] Turchi C.S., Vidal J., Bauer M. (2018). Molten salt power towers operating at 600–650 °C: Salt selection and cost benefits. Sol. Energy.

[20] Nunes V.M.B., Lourenço M.J.V., Santos F.J.V., Nieto de Castro C.A. (2019). Molten alkali carbonates as alternative engineering fluids for high temperature applications. Appl. Energy.

[21] Fereres S., Prieto C., Giménez-Gavarrell P., Rodríguez A., Sánchez-Jiménez P.E., Pérez-Maqueda L.A. (2018). Molten carbonate salts for advanced solar thermal energy power plants: Cover gas effect on fluid thermal stability. Sol. Energy Mater. Sol. Cells.

[22] Prieto C., Fereres S., Ruiz-Cabañas F.J., Rodriguez-Sanchez A., Montero C. (2020). Carbonate molten salt solar thermal pilot facility: Plant design, commissioning and operation up to 700 °C. Renew. Energy.

[23] Sarvghad M., Steinberg T.A., Will G. (2017). Corrosion of steel alloys in eutectic NaCl+Na2CO3 at 700 °C and Li2CO3 + K2CO3 + Na2CO3 at 450 °C for thermal energy storage. Sol. Energy Mater. Sol. Cells.

[24] Morales M., Gordon S., Fernández-Arana Ó., García-Marro F., Mateo A., Llanes L., Fargas G. (2022). Duplex Stainless Steels for Thermal Energy Storage: Characterization of Oxide Scales Formed in Carbonate Salts at 500 °C. Metals.

[25] Morales M., Cabezas L., Castro-Alloca M., Fargas G., Llanes L., Mateo A. (2022). Corrosion Evaluation of Austenitic and Duplex Stainless Steels in Molten Carbonate Salts at 600 °C for Thermal Energy Storage. Metals.

[26] Sarvghad M., Will G., Steinberg T.A. (2017). Corrosion of Inconel 601 in molten salts for thermal energy storage. Sol. Energy Mater. Sol. Cells.

[27] Sah P.P. (2020). Corrosion of 304 stainless steel in carbonates melt—A state of enhanced dissolution of corrosion products. Corros. Sci..

[28] de Miguel M.T., Encinas-Sánchez V., Lasanta M.I., García-Martín G., Pérez F.J. (2016). Corrosion resistance of HR3C to a carbonate molten salt for energy storage applications in CSP plants. Sol. Energy Mater. Sol. Cells.

[29] Aljaerani H.A., Samykano M., Saidur R., Pandey K.K., Kadirgama K. (2021). Nanoparticles as molten salts thermophysical properties enhancer for concentrated solar power: A critical review. J. Energy Storage.

[30] Grosu Y., Nithiyanantham U., Zaki A., Faik A. (2018). A simple method for the inhibition of the corrosion of carbon steel by molten nitrate salt for thermal storage in concentrating solar power applications. npj Mater. Degrad..

[31] Grosu Y., Anagnostopoulos A., Navarro M.E., Ding Y., Faik A. (2020). Inhibiting hot corrosion of molten Li2CO3-Na2CO3-K2CO3 salt through graphitization of construction materials for concentrated solar power. Sol. Energy Mater. Sol. Cells.

[32] Raiman S.S., Mayes R.T., Kurley J.M., Parrish R., Vogli E. (2019). Amorphous and partially-amorphous metal coatings for corrosion resistance in molten chloride salt. Sol. Energy Mater. Sol. Cells.

[33] González-Fernández L., Serrano Á., Palomo E., Grosu Y. (2023). Nanoparticle-based anticorrosion coatings for molten salts applications. J. Energy Storage.

[34] Morales M., Rezayat M., Mateo A. (2024). Amorphous Carbon Film as a Corrosion Mitigation Strategy for Stainless Steel in Molten Carbonate Salts for Thermal Energy Storage Applications. Materials.

[35] Abu-warda N., Bedmar J., García-Rodriguez S., Utrilla M.V., Torres B., Rams J. (2024). Impact of molten salts composition on the corrosion behavior of NiMoCr and CoNiCrAl coatings on L-PBF 316L stainless steel for CSP plants. Surf. Coat. Technol..

[36] Agüero A., García de Blas F.J., García M.C., Muelas R., Román A. (2001). Thermal spray coatings for molten carbonate fuel cells separator plates. Surf. Coat. Technol..

[37] Kondaiah P., Pitchumani R. (2021). Fractal textured surfaces for high temperature corrosion mitigation in molten salts. Sol. Energy Mater. Sol. Cells.

[38] González-Fernández L., Anagnostopoulos A., Karkantonis T., Bondarchuk O., Dimov S., Chorążewski M., Ding Y., Grosu Y. (2022). Laser-induced carbonization of stainless steel as a corrosion mitigation strategy for high-temperature molten salts applications. J. Energy Storage.

[39] Morales M., Rezayat M., Mateo A. (2024). Enhancing the corrosion resistance of 2205 duplex stainless steel in molten carbonate salts by laser-surface texturing. J. Energy Storage.

[40] Rezayat M., Morales M., Moradi M., Mateo A. (2024). Laser wobbling surface texturing of AISI 301LN steel for enhancement of the corrosion resistance at high temperature. Opt. Laser Technol..

[41] Rezayat M., Morales M., Moradi M., Mateo A. (2025). Influence of laser heat input on the corrosion mitigation of laser textured AISI 301LN steel in molten carbonate salts. Opt. Laser Technol..

[42] Rezayat M., Morales M., Zahrani E.G., Moradi M., Azarhoushang B., Mateo A. (2025). Effect of repetition passes in the laser surface texturing of AISI 301LN steel on the anticorrosion properties in molten carbonate salts. Materialia.

[43] Xie Z., Liu B., Fu A., Li K., Cao Y., Zhou H., Peng J., Liu Y. (2024). Combating Cl^−^ and SO_4_^2−^—Induced molten salt corrosion by laser cladding FeCrNiMoAl high-entropy alloy coating. Surf. Coat. Technol..

[44] Fernández A.G., Muñoz-Sánchez B., Nieto-Maestre J., García-Romero A. (2019). High temperature corrosion behavior on molten nitrate salt-based nanofluids for CSP plants. Renew. Energy.

[45] Nithiyanantham U., Grosu Y., González-Fernández L., Zaki A., Igartua J.M., Faik A. (2019). Corrosion aspects of molten nitrate salt-based nanofluids for thermal energy storage applications. Sol. Energy.

[46] Gao Q., Lu Y., Wang Y., Wu Y., Zhang C., Wang Y. (2024). Electrochemical study on the corrosion behavior of 316L stainless steel in quaternary nitrate molten salt nanofluids for thermal energy storage applications. J. Energy Storage.

[47] Nieto-Maestre J., Muñoz-Sánchez B., Fernández A.G., Faik A., Grosu Y., García-Romero A. (2020). Compatibility of container materials for Concentrated Solar Power with a solar salt and alumina based nanofluid: A study under dynamic conditions. Renew. Energy.

[48] Nithiyanantham U., Grosu Y., González-Fernández L., Zaki A., Igartua J.M., Faik A. (2019). Development of molten nitrate salt based nanofluids for thermal energy storage application: High thermal performance and long storage components life-time. AIP Conf. Proc..

[49] Nithiyanantham U., Grosu Y., Anagnostopoulos A., Carbó-Argibay E., Bondarchuk O., González-Fernández L., Zaki A., Igartua J.M., Navarro M.E., Ding Y. (2019). Nanoparticles as a high-temperature anticorrosion additive to molten nitrate salts for concentrated solar power. Sol. Energy Mater. Sol. Cells.

[50] Grosu Y., Udayashankar N., Bondarchuk O., González-Fernández L., Faik A. (2018). Unexpected effect of nanoparticles doping on the corrosivity of molten nitrate salt for thermal energy storage. Sol. Energy Mater. Sol. Cells.

[51] Piquot J., Nithiyanantham U., Grosu Y., Faik A. (2019). Spray-graphitization as a protection method against corrosion by molten nitrate salts and molten salts based nanofluids for thermal energy storage applications. Sol. Energy Mater. Sol. Cells.

[52] Shin D., Banerjee D. (2010). Effects of silica nanoparticles on enhancing the specific heat capacity of carbonate salt eutectic (work in progress). Int. J. Struct. Chang. Sol..

[53] Wu Y.T., Ren N., Wang T., Ma C.F. (2011). Experimental study on optimized composition of mixed carbonate salt for sensible heat storage in solar thermal power plant. Sol. Energy.

[54] Tao Y.B., Lin C.H., He Y.L. (2015). Preparation and thermal properties characterization of carbonate salt/carbon nanomaterial composite phase change material. Energy Convers. Manag..

[55] Sang L., Liu T. (2017). The enhanced specific heat capacity of ternary carbonates nanofluids with different nanoparticles. Sol. Energy Mater. Sol. Cells.

[56] Grosu Y., Anagnostopoulos A., Balakin B., Krupanek J., Navarro M.E., González-Fernández L., Ding Y., Faik A. (2021). Nanofluids based on molten carbonate salts for high-temperature thermal energy storage: Thermophysical properties, stability, compatibility and life cycle analysis. Sol. Energy Mater. Sol. Cells.

[57] Borode A., Tshephe T., Olubambi P. (2024). A critical review of the thermophysical properties and applications of carbon-based hybrid nanofluids in solar thermal systems. Front. Energy Res..

[58] Rabani R., Hosseinian Naeini K. (2025). Molten Salt Nanofluids for High-Temperature Thermal Energy Storage: Advances, Mechanisms, and Challenges. Sustain. Energy Artif. Intell..

[59] Gurgenc E., Öztop H.F., Yamaç H.İ., Canbay C.A., Şenocak Ş.M., Özabacı M., Gurgenc T., Gür M. (2025). B4C-based nanoenhancement on the thermophysical and stability performance of solar salt: A novel approach for high-temperature TES applications. Case Stud. Therm. Eng..

[60] Jamei M., Alkouh A.B., Karbasi M., Yaseen Z.M. (2024). Thermo-physical properties estimation of an oil-based hybrid nanofluid: Application of a new hybrid neurocomputing approach. J. Therm. Anal. Calorim..

[61] Ji C., Yang X., Xu H., Yang Z., Xie J. (2025). Molecular insight into further enhancement of thermal conductivity and heat capacity of K2CO3-SiO2 molten salt nanofluids by oxygen vacancy defects in SiO2 nanoparticles. Int. J. Heat Mass Transf..

[62] Altwarah Q., Abir F.M., Prince C., Shin D. (2025). Exploring the impact of nanostructures on specific heat in nanoparticle-doped carbonate salts via MD simulations. Sol. Energy Mater. Sol. Cells.

[63] Huang Z., Hu Z., Li Q., Qiu Y. (2025). Property regulations of binary alkali carbonates by SiO2 nanoparticles for high-temperature thermal energy storage. Sol. Energy Mater. Sol. Cells.

[64] Pandian A., Boobalan C. (2025). Ternary hybrid nanofluids: Synthesis, thermal properties, machine learning insights, and emerging applications. Multiscale Multidiscip. Model. Exp. Des..

[65] Mirahmad A., Kumar R.S., Doldán B.P., Rios C.P., Díez-Sierra J. (2025). Beyond Thermal Conductivity: A Review of Nanofluids for Enhanced Energy Storage and Heat Transfer. Nanomaterials.

[66] Schuller M., Little F., Malik D., Betts M., Shao Q., Luo J., Zhong W., Shankar S., Padmanaban A. (2012). Molten Salt-Carbon Nanotube Thermal Energy Storage for Concentrating Solar Power Systems Final Report.

[67] Frangini S., Loreti S. (2007). The role of alkaline-earth additives on the molten carbonate corrosion of 316L stainless steel. Corros. Sci..

[68] Yang X., Jiang W., Ji C., Wang Q. (2022). Experimental study on heat storage and corrosion properties of ternary carbonate salt-based ZnO nanofluids for solar thermal energy storage. J. Therm. Anal. Calorim..

[69] Fernández A.G., Pineda F., Walczak M., Cabeza L.F. (2019). Corrosion evaluation of alumina-forming alloys in carbonate molten salt for CSP plants. Renew. Energy.

[70] Morales M., Rezayat M., Fargas G., Mateo A. (2024). Mitigating the corrosion of AISI 301LN steel in molten carbonate salts by doping with alumina nanoparticles for thermal energy storage applications. Sol. Energy Mater. Sol. Cells.

[71] Kaladhar M., Venkata Subbaiah K., Srinivasa Rao C.H. (2012). Machining of austenitic stainless steels—A review. Int. J. Mach. Mach. Mater..

[72] Morales M., Chimenos J.M., Fernández A.I., Segarra M. (2014). Materials selection for superheater tubes in municipal solid waste incineration plants. J. Mater. Eng. Perform..

[73] Holappa L., Kekkonen M., Jokilaakso A., Koskinen J. (2021). A Review of Circular Economy Prospects for Stainless Steelmaking Slags. J. Sustain. Metall..

[74] Mateo A., Fargas G., Zapata A. (2012). Martensitic Transformation during Fatigue Testing of an AISI 301LN Stainless Steel. IOP Conf. Ser. Mater. Sci. Eng..

[75] Martelo D.F., Mateo A.M., Chapetti M.D. (2015). Fatigue crack growth of a metastable austenitic stainless steel. Int. J. Fatigue.

[76] Rezayat M., Moradi M., Mateo A. (2023). Nanosecond pulsed laser surface processing of AISI 301LN steel: Effect on surface topography and mechanical properties. Int. J. Adv. Manuf. Technol..

[77] Rezayat M., Karamimoghadam M., Moradi M., Casalino G., Roa Rovira J.J., Mateo A. (2023). Overview of Surface Modification Strategies for Improving the Properties of Metastable Austenitic Stainless Steels. Metals.

[78] (2017). Standard Practice for Preparing, Cleaning, and Evaluating Corrosion Test Specimens.

[79] Oliver W.C., Pharr G.M. (2004). Measurement of hardness and elastic modulus by instrumented indentation: Advances in understanding and refinements to methodology. J. Mater. Res..

[80] Morales M., Chimenos J.M., Espiell F., Segarra M. (2014). The effect of temperature on mechanical properties of oxide scales formed on a carbon steel in a simulated municipal solid waste incineration environment. Surf. Coat. Technol..

[81] Tan L., Yang Y., Allen T.R. (2006). Oxidation behavior of iron-based alloy HCM12A exposed in supercritical water. Corros. Sci..

[82] Biedenkopf P., Spiegel M., Grabke H.J. (1997). The corrosion behavior of Fe-Cr alloys containing Co, Mn, and/or Ni and of a Co-base alloy in the presence of molten (Li,K)-carbonate. Mater. Corros..

[83] Soleimani Dorcheh A., Durham R.N., Galetz M.C. (2016). Corrosion behavior of stainless and low-chromium steels and IN625 in molten nitrate salts at 600 °C. Sol. Energy Mater. Sol. Cells.

[84] Bell S., Steinberg T., Will G. (2019). Corrosion mechanisms in molten salt thermal energy storage for concentrating solar power. Renew. Sustain. Energy Rev..

[85] Hong Y., Zhou C., Zheng Y., Zhang L., Zheng J., Chen X. (2019). Dependence of strain rate on hydrogen-induced hardening of austenitic stainless steel investigated by nanoindentation. Int. J. Hydrogen Energy.

[86] England J., Uddin M.J., Ramirez-Cedillo E., Karunarathne D., Nasrazadani S., Golden T.D., Siller H.R. (2022). Nanoindentation Hardness and Corrosion Studies of Additively Manufactured 316L Stainless Steel. J. Mater. Eng. Perform..

[87] Zhang X., Yang D., Jia Y., Wang G. (2023). Microstructure and Nanoindentation Behavior of FeCoNiAlTi High-Entropy Alloy-Reinforced 316L Stainless Steel Composite Fabricated by Selective Laser Melting. Materials.

[88] Hajiannia I., Shamanian M., Kasiri M. (2013). Microstructure and mechanical properties of AISI 347 stainless steel/A335 low alloy steel dissimilar joint produced by gas tungsten arc welding. Mater. Des..

[89] Schütze M., Malessa M., Renusch D., Tortorelli P.F., Wright I.G., Dooley R.B. (2006). Mechanical Properties and Adherence of Oxide Scales. Mater. Sci. Forum.

[90] Evans H.E. (1995). Stress effects in high temperature oxidation of metals. Int. Mater. Rev..

[91] Camacho I., Chen Q., González-Fernández L., Bondarchuk O., Bartolomé L., Jiang Z., Ding Y., Grosu Y. (2022). On the anticorrosion mechanism of molten salts based nanofluids. Sol. Energy Mater. Sol. Cells.

[92] Ibrahim A., Peng H., Riaz A., Abdul Basit M., Rashid U., Basit A. (2021). Molten salts in the light of corrosion mitigation strategies and embedded with nanoparticles to enhance the thermophysical properties for CSP plants. Sol. Energy Mater. Sol. Cells.

[93] Gao Q., Lu Y., Yu Q., Wu Y., Zhang C., Zhi R. (2022). High-temperature corrosion behavior of austenitic stainless steel in quaternary nitrate molten salt nanofluids for concentrated solar power. Sol. Energy Mater. Sol. Cells.

